# IL-2 immunotherapy in chronically SIV-infected Rhesus Macaques

**DOI:** 10.1186/1743-422X-9-220

**Published:** 2012-09-28

**Authors:** Julie Garibal, Mireille Laforge, Ricardo Silvestre, Shahul Mouhamad, Laure Campillo-Gimenez, Yves Lévy, Jérôme Estaquier

**Affiliations:** 1 INSERM U955 Equipe 16, Institut Mondor de Recherche Biomédicale, Créteil, F-94010, France; 2 Université Paris-Est, Faculté de Médecine, UMR-S 955, Créteil, F-94010, France; 3 Assistance Publique-Hôpitaux de Paris (AP-HP), Groupe Henri-Mondor Albert-Chenevier, service d’immunologie clinique, Créteil, F-94010, France; 4 CNRS FRE 3235, Université Paris Descartes, Paris, France; 5 Université Laval, Centre de recherche en Infectiologie, Québec, Canada; 6 Instituto de Biologia Molecular e Celular, Universidade do Porto, Porto, Portugal

**Keywords:** SIV, IL-2 immunotherapy, T cells, Apoptosis, Fas, Treg

## Abstract

**Background:**

Despite inducing a sustained increase in CD4+ T cell counts, intermittent recombinant IL-2 (rIL-2) therapy did not confer a better clinical outcome in HIV-infected patients enrolled in large phase III clinical trials ESPRIT and SILCAAT. Several hypotheses were evoked to explain these discrepancies. Here, we investigated the impact of low and high doses of IL-2 in Rhesus macaques of Chinese origin infected with SIVmac251 in the absence of antiretroviral therapy (ART).

**Results:**

We demonstrated that rIL-2 induced a dose dependent expansion of CD4+ and CD8+ T cells without affecting viral load. rIL-2 increased CD4 and CD8 Treg cells as defined by the expression of CD25^high^FoxP3^+^CD127^low^. We also showed that rIL-2 modulated spontaneous and Fas-mediated CD4^+^ and CD8^+^ T cell apoptosis. The higher dose exhibited a dramatic pro-apoptotic effect on both CD4^+^ and CD8^+^ T cell populations. Finally, all the animals treated with rIL-2 developed a wasting syndrome in the month following treatment simultaneously to a dramatic decrease of circulating effector T cells.

**Conclusion:**

These data contribute to the understanding of the homeostatic and dosage effects of IL-2 in the context of SIV/HIV infection.

## Background

Progressive decrease in the number of CD4^+^ T lymphocytes is the hallmark of HIV (Human Immunodeficiency Virus) or SIV (Simian Immunodeficiency Virus) infection. During this chronic infection, CD4^+^ T cell loss, generalized immune activation and increased susceptibility to apoptosis result in impaired T cell homeostasis and subsequent development of opportunistic infections [1]. Mechanisms leading to CD4^+^ T cell depletion are not completely understood yet but viral replication plays a key role in this process. Highly Active Antiretroviral therapy (HAART), by allowing virological suppression, significant rise in CD4^+^ T cell counts and decrease in apoptosis sensitivity, has dramatically improved clinical outcome for HIV patients. However, this treatment partially fails to normalize HIV/SIV-associated immune dysregulation and, in addition, implies risk of drug resistance and long terms toxicity [2,3]. This has led to evaluate therapeutic strategies that could reinforce HAART effect or even delay the initiation of antiretroviral treatment. The best studied of these therapeutic approaches is intermittent administration of interleukin-2 (IL-2).

IL-2 is an autocrine T cell growth factor mainly produced by activated T cells and implicated in the homeostasis and differentiation of Th1, Th2, Th17 and regulatory T cell subsets. In HIV infection, due to progressive loss of CD4^+^ T cells, levels of IL-2 are reduced. This progressive decrease of IL-2 producing CD4^+^ T cells has been directly correlated with activation state and susceptibility to apoptosis of these cells [4-7]. Moreover, IL-2 was found to improve survival of HIV patients’ T cells *ex vivo* through the upregulation of the anti-apoptotic protein Bcl-2 [8-10]. Partial restoration of IL-2 producing CD4^+^ T cell pool is observed following treatment with HAART [11]. On the other hand, IL-2 has also been shown to play a key role in pro-apoptotic processes. Indeed, IL-2 is required for priming T cells to undergo Activation-Induced Cell Death (AICD), a process that is Fas-mediated and serves as a feedback regulation of clonal expansion [12]. Mice deficient for IL-2 develop lymphoproliferation and autoimmune diseases rather than immunodeficiencies [13]. IL-2 has also been shown to induce directly the expression of programmed death (PD)-1 molecule and its ligand PD-L1, negative regulatory members of the B7 family that play an important role in peripheral tolerance [14]. In addition, IL-2, by favoring viral replication *in vitro*, may induce apoptosis in productively HIV/SIV-infected T cells [15]. IL-2 immunotherapy has been extensively studied in phase I/II and III studies. Intermittent treatment with IL-2 induces significant and sustained rise in CD4^+^ T cell counts [16-19]. In particular, recombinant IL-2 therapy raised naïve and central memory CD4^+^ T cells [20]. Analysis of T cell populations, using BrdU labeling in HIV patients treated with IL-2, revealed a decreased sensitivity to apoptosis of CD4^+^ T cells [21]. In addition, *in vivo* administration of IL-2 was shown to induce expansion of a CD4 T cell population that expressed CD45RO, CD25 and FoxP3 at high levels but exerted weak suppressive activity and were, thus, not considered as regulatory T cells (Treg) [22]. On the other hand, a recent study highlighted this point, showing that IL-2 therapy led to expansion of two distinct CD4^+^ CD25^high^ T cell populations that could suppress effector cells proliferation *in vitro*[23]. This observation may partially explain the disappointing results of the large phase III trials SILCAAT and ESPRIT. In these studies, although the average CD4^+^ T cell counts were significantly higher in IL-2 arms, IL-2 plus HAART yielded no added clinical benefit as compared with HAART alone [24].

Here, in order to better understand effects of IL-2 on T lymphocytes populations, we used Rhesus macaques of Chinese origin infected with SIVmac251 treated with human recombinant IL-2 (rIL-2) in the absence of Highly Active Anti-Retroviral Therapy (HAART). We showed that IL-2 immunotherapy exerted dose dependent effects on several immunological parameters without affecting viral load. Treatment with rIL-2 increased CD4^+^ and CD8^+^ T cell counts in a dose dependent manner and induced CD4 and, more interestingly, CD8 Treg as defined by the expression of CD25^high^FoxP3^+^CD127^low^. We also showed that IL-2 had a dual effect on spontaneous and Fas-induced apoptosis of CD4^+^ and CD8^+^ T cells with low dose exerting an anti-apoptotic effect whereas high dose exhibited a dramatic pro-apoptotic effect on both CD4^+^ and CD8^+^ T cell populations. Finally, all the animals treated with rIL-2 developed a wasting syndrome in the month following treatment.

## Results

### Human rIL-2 transiently increases CD4 and CD8 T cell counts in a dose-dependent manner

Rhesus macaques infected with SIVmac251 at steady state of chronic phase (four months post infection), received rIL-2 either at low dose (1.4x10^5^ UI/kg body weight) (#056212 and #056216) or at higher dose (6.10^5^ UI/kg body weight) (#056230 and #056238) subcutaneously daily for 5 days.

**Figure 1:**
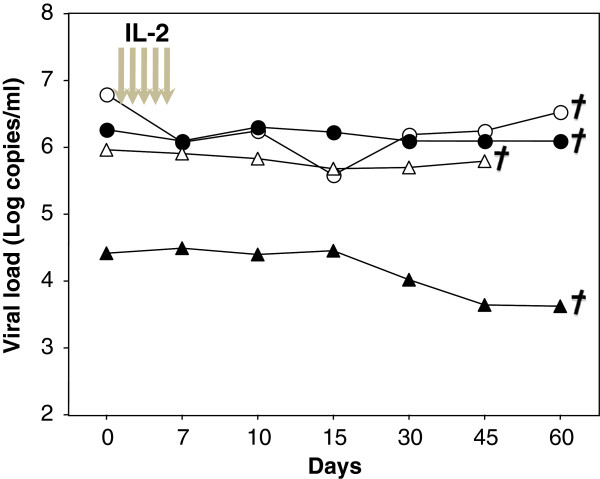
**Human rIL**-**2 does not affectviral load.** Four Rhesus macaques were infected with the pathogenic SIVmac251 strain and then treated during chronic phase with human rIL-2 (low dose: О,Δ or high dose: ●,▴). Each symbol represents one monkey. Viral load (copies/ml) was quantified by RT-PCR at the day of treatment and thereafter. Euthanasia date is represented.

Hematological and physiological parameters such as platelet, erythrocyte and neutrophil counts, hematocrit, temperature, weight were followed and remained unchanged during IL-2 cycle (see Additional file 1). Viral load was followed in all 4 animals and was not affected by rIL-2 treatment at either high or low dose (Figure 1). However, after one month, all treated animals developed a wasting syndrome with weight loss and diarrhea and were euthanized. Therefore, for obvious ethical reasons, no more animals were included in this protocol.

**Figure 2:**
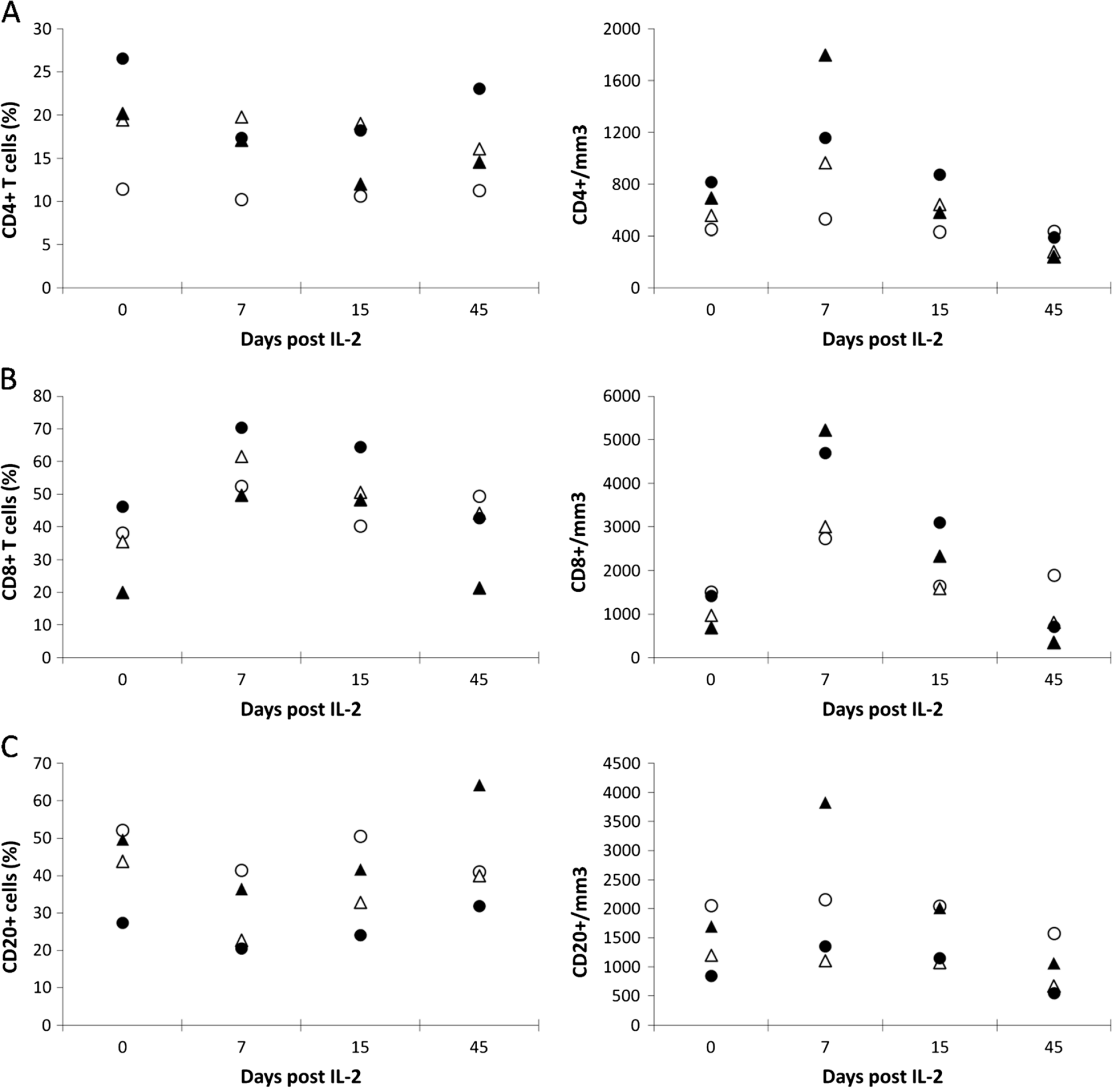
**Human rIL**-**2 transiently increases CD4**^+ ^**and CD8**^+ ^**T cell counts ina dose**-**dependent manner.** Percentages and counts of peripheral blood (**A**) CD4^+^, (**B**) CD8^+^ T cells and (**C**) CD20^+^ B cells were measured at day 0, 7, 15 and 45 after IL-2-treatment (low dose: О,Δ or high dose: ●,▴).

Treatment with rIL-2 increased dramatically CD4^+^ and CD8^+^ T cell counts in a dose-dependent manner while CD20^+^ B cell counts remained unchanged (Figure 2). In animals treated with low doses, absolute peripheral CD4^+^ T cell counts rose from 450 and 560 cells/mm^3^ (before treatment) to 530 cells/mm^3^ and 970 cells/mm^3^ (at day 7) while those treated at the highest dose, experienced an increase from 690 and 815 to 1800 and 1160 cells/mm^3^, respectively.

In addition, the effect of rIL-2 treatment on CD8 T cell counts was even more pronounced with 2.6 and 1.4 fold increases from baseline in the two animals treated at low dose (Δcounts = +1235 and +2045 cells/mm^3^) and 7.6 and 3.3 fold increases in those treated at highest doses (Δcounts = +4545 and +3285 cells/mm^3^). CD4^+^ and CD8^+^ changes were transient and T cell counts returned to baseline at day 15. In control SIV-infected Rhesus macaques who received a placebo (NaCl) (n = 5), CD4 and CD8 T cell counts remained stable during the same period of time (data not shown).

At day 45, all animals experienced a drastic CD4 T cell lymphopenia (Δcounts = −20 and −290 cells/mm^3^ in animals treated with low dose and −450 and −425 cells/mm^3^ and in animals treated with high dose) whereas CD8 lymphopenia was only observed in recipients of highest dose of rIL-2 (Δcounts = −335 and −695 cells/mm3) (Figure 2).

### Human rIL-2 has a differential and dose-dependent effect on CD4 and CD8 T cell subpopulations

**Figure 3:**
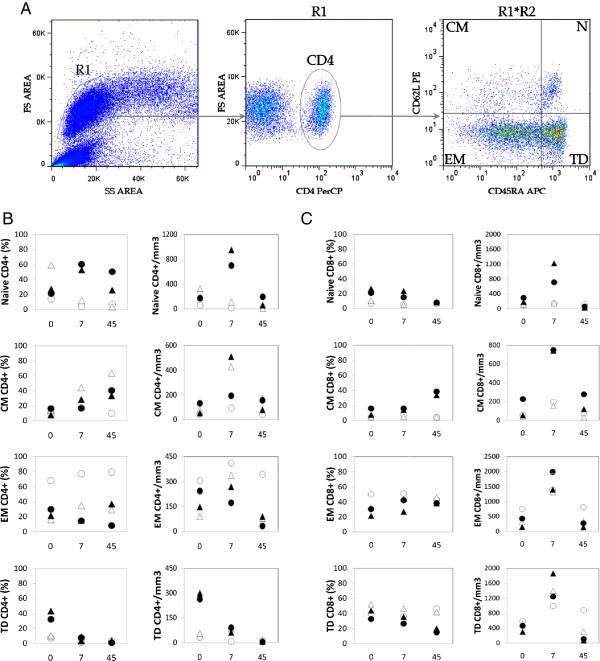
**Human rIL**-**2 has a differential**, **dose**-**dependent effect on CD4**^+ ^**and CD8**^+ ^**T cell subsets.** (**A**) Gating strategy to identify naive (N), central memory (CM), effector memory (EM) and terminal differenciated (TD) T lymphocytes. Cells were first gated on side and forward scatter parameters (gate R1) and further gated on CD4^+^ (or CD8^+^) T cells (gate R2). Thereafter, CD4^+^ (or CD8^+^) T cell subsets were analyzed for the expression of CD45RA and CD62L molecules. CD45RA^+^CD62L^+^ were identified as naive, CD45RA^-^CD62L^+^ as central memory (CM), CD45RA^-^CD62L^-^ as effector memory (EM) and CD45RA^+^CD62L- as terminal differentiated (TD) T cells. (**B**) Percentages and counts of each CD4^+^ T cell subpopulation were followed in IL-2-treated animals - low dose (ОΔ) or high dose (●▴) (**C**) Similarly, percentages and counts of each CD8^+^ T cell subpopulation were followed in IL-2-treated animals - low dose (ОΔ) or high dose (●▴).

We next sought to determine the effects of rIL-2 on T cell subpopulations defined by the expression of CD45RA and CD62L. In this analysis, CD45RA^+^CD62L^+^ were identified as naïve T cells, CD45RA^-^CD62L^+^ were identified as central memory T cells, CD45RA^-^CD62L^-^ were identified as effector memory T cells and CD45RA^+^CD62L^-^ were identified as terminal differentiated T cells (Figure 3A). No significant differences between controls (n = 5) and IL-2 treated animals (n = 4) were seen at baseline (data not shown). High dose of rIL-2 led to a dramatic increase of frequency and absolute counts of naïve CD4 T cell at day 7 (Δ% = +25.9 and 39.4%, Δcounts = +765 and 530 cells/mm^3^). In contrast, in animals treated with low doses of IL-2, percentages and absolute counts of naïve CD4^+^ T cells decreased at day 7 after treatment (Δ% = −10.2 and −47.3%, Δcounts = −43 and −220 cells/mm^3^).

As seen in Figure 3B, low dose of rIL-2 increased absolute counts and percentages of effector memory populations at day 7 from 68 and 16% to 77 and 35%. This effect was even more pronounced in central memory CD4^+^ T cells as this subpopulation accounted from 10 and 14% to 18 and 44% , respectively, of circulating CD4^+^ T cells. Animals treated with high dose of rIL-2 also showed an increase in proportion of central memory CD4^+^ T cells (Δ% = +20.3 and 3.8%) but not effector memory T cells. In addition, high dose of rIL-2 also exerted a “long term” effect on central memory CD4^+^ T cells as proportion of this subpopulation increased by 25.5 and 24.1% in the two animals at day 45.

Finally, terminally differentiated CD4^+^ T cells were drastically depleted at day 45 after treatment, whatever the dose of rIL-2 used, with absolute counts remaining under 10 cells/mm^3^ corresponding to 5.3% and 35.7% decreases for low and high dose respectively (Figure 3B).

High and low dose of rIL-2 therapy exhibited only modest changes in distribution of CD8^+^ T populations at day 7. Percentages of effector memory CD8 T cells increased by 5.3 and 12.1% in low and and 11.1 and 2.2% in high dose treated animals. Similarly to its effect on CD4 T cell populations, high rIL-2 dose seemed to exert a more long term effect on central memory CD8 T cell pool as it increased from 7.8 and 16.1% before treatment to 33.7 and 38.4% at day 45.

Thus, in SIV-infected Rhesus macaques, rIL-2 treatment had a differential, dose-dependent effect on T cell populations, high dose allowing expansion of both naïve and central memory CD4 T cell subpopulations and low dose only exerting its effect on CD4 T cell memory subsets (central and effector). High and low dose both seemed to induce a drastic depletion of terminally differentiated CD4^+^ T cells at day 45 after treatment. The impact of rIL-2 treatment on distribution of CD8^+^ T cells was restricted to effector memory subpopulation whatever the dose injected with high dose having a slight effect on long term central memory CD8 T cells expansion.

### Human rIL-2 transiently increases peripheral blood CD4^+^ and CD8^+^ tregs in a dose-dependent manner

**Figure 4:**
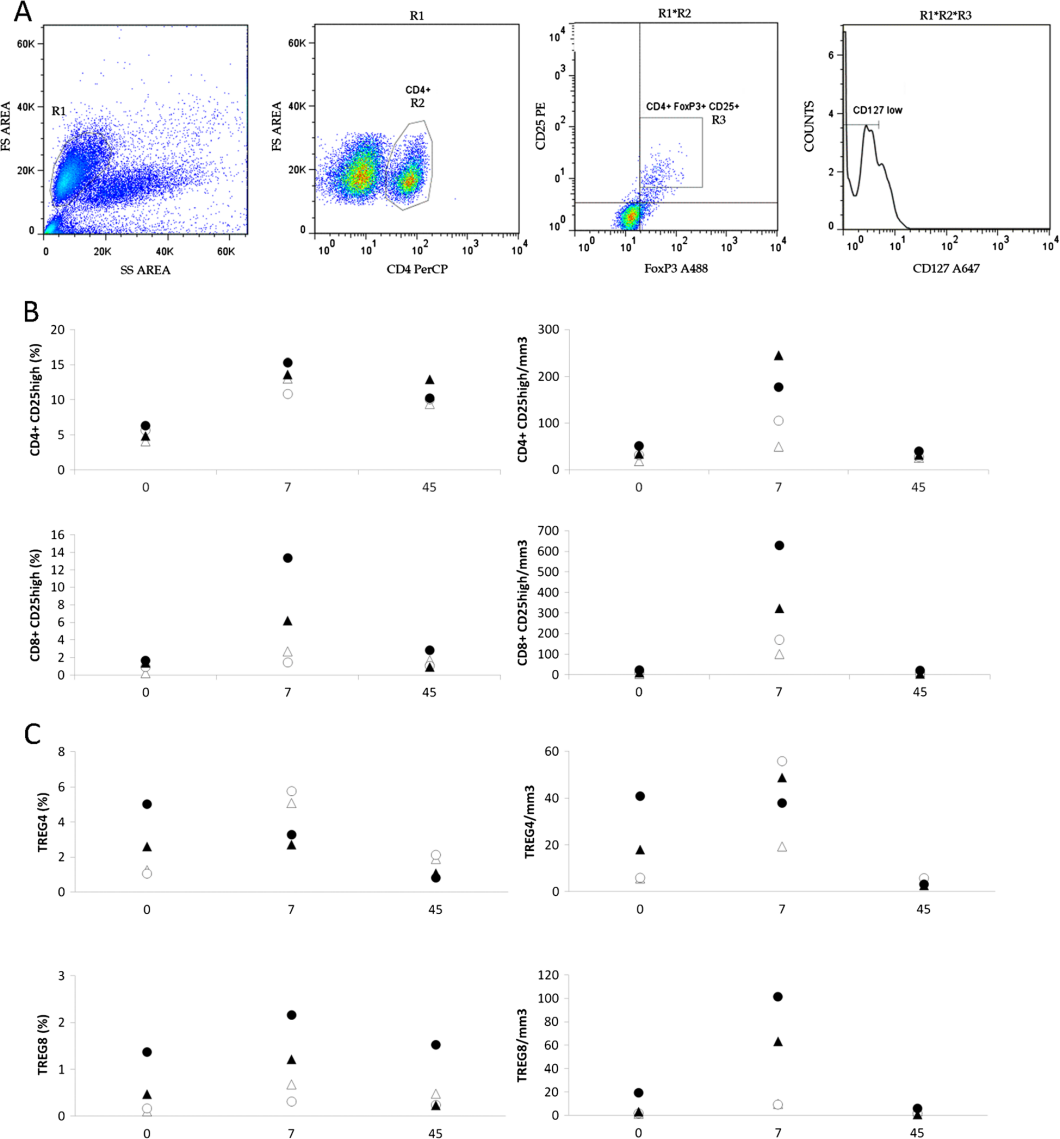
**Human rIL**-**2 transiently increases peripheralblood CD4**^+ ^**and CD8**^+ ^**Tregs in a dose**-**dependent manner.** (**A**) Gating strategy for the identification of Treg cells. Cells were first gated on side and forward scatter parameters (gate R1) and further gated on CD4^+^ T cells (gate R2) to analyze CD25 and FoxP3 expression. CD4^+^FoxP3^+^CD25^high^ or CD8^+^FoxP3^+^CD25^high^ (R3) were then analyzed for CD127 expression. CD4^+^FoxP3^+^CD25^high^CD127^low^ or CD8^+^FoxP3^+^CD25^high^CD127^low^ were identified as CD4 or CD8 Tregs respectively. (**B**) Percentages and counts of peripheral blood CD4^+^CD25^high^ and CD8^+^CD25 ^high^ cells in IL-2-treated macaques (low dose, #056212, #056216 – high dose, #056230, #056238) at days 0, 7 and 45 after IL-2 treatment. (**C**) Percentages and counts of CD4 or CD8 Tregs in IL-2-treated macaques (low dose #056212, #056216 – high dose #056230, #056238) at day 0, 7 and 45 after IL-2 treatment.

We evaluated the frequencies of CD4^+^ CD25^high^ and CD8^+^ CD25^high^ in IL-2 recipients (Figure 4B). Treatment with rIL-2 induced a transient increase in CD4^+^ CD25^high^ (Δ% = +8.9 and +5.2%, and +8.7 and +9% at low and high doses, respectively) and CD8^+^ CD25^high^ populations (Δ% = +3.5 and +6.6%, and +4.8 and +11.8% at low and high doses, respectively). At day 45, frequency of CD8^+^ CD25^high^ population returned to baseline levels whereas CD4^+^ CD25^high^ subset remained higher than pretreatment values (Δ% = +5.3 and +4.5% and +8.15 and +4% in animals treated with low and high doses, respectively).

As CD25^high^ cells could be activated cells or Tregs, we defined CD4^+^ Tregs as CD4^+^ FoxP3^+^ CD25^high^ CD127^low^ cells and CD8^+^ Tregs as CD8^+^ FoxP3^+^ CD25^high^ CD127^low^ (Figure 4A). As seen in Figure 4C (top panel), low dose of rIL-2 provoked a robust increase in CD4^+^ Treg subset frequency (Δ% = +2.5 and +4.8%) and counts at day 7. This frequency then returned to basal levels at day 45. When macaques were treated with high dose of rIL-2, no increase in frequency of CD4^+^ Treg subset could be observed at day 7 (Δ% = −0.1 and −1.9%). In contrast, peripheral CD8^+^ Treg population was expanded at day 7 after rIL-2 injection, whatever the dose used (Figure 4C – bottom panel) by a 1.5 to 6.6 fold.

### Human rIL-2 transiently increases proliferation and activation of CD4^+^ and CD8^+^T cells

**Figure 5:**
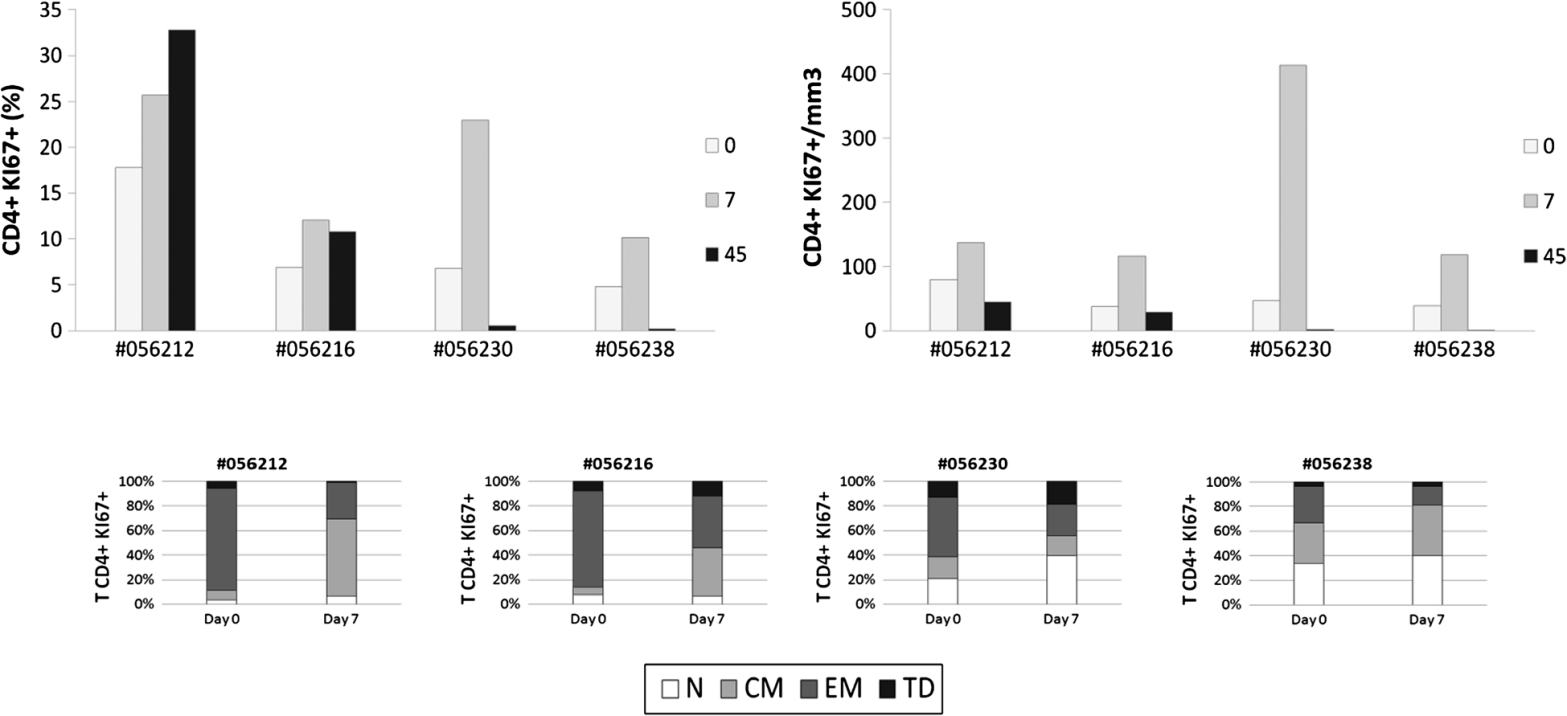
**Human rIL**-**2 transiently increases proliferationof CD4**^+ ^**T cell subset.** Ki67 expression was measured intracellularly in CD4^+^ T cells (top panel). Ki67 expression was then assessed in each CD4^+^ T cell subset (N, CM, EM or TD) at days 0 and 7 after IL-2 treatment (bottom panel). IL-2-treated macaques were #056212, #056216 (low dose) and #056230, #056238 (high dose).

**Figure 6:**
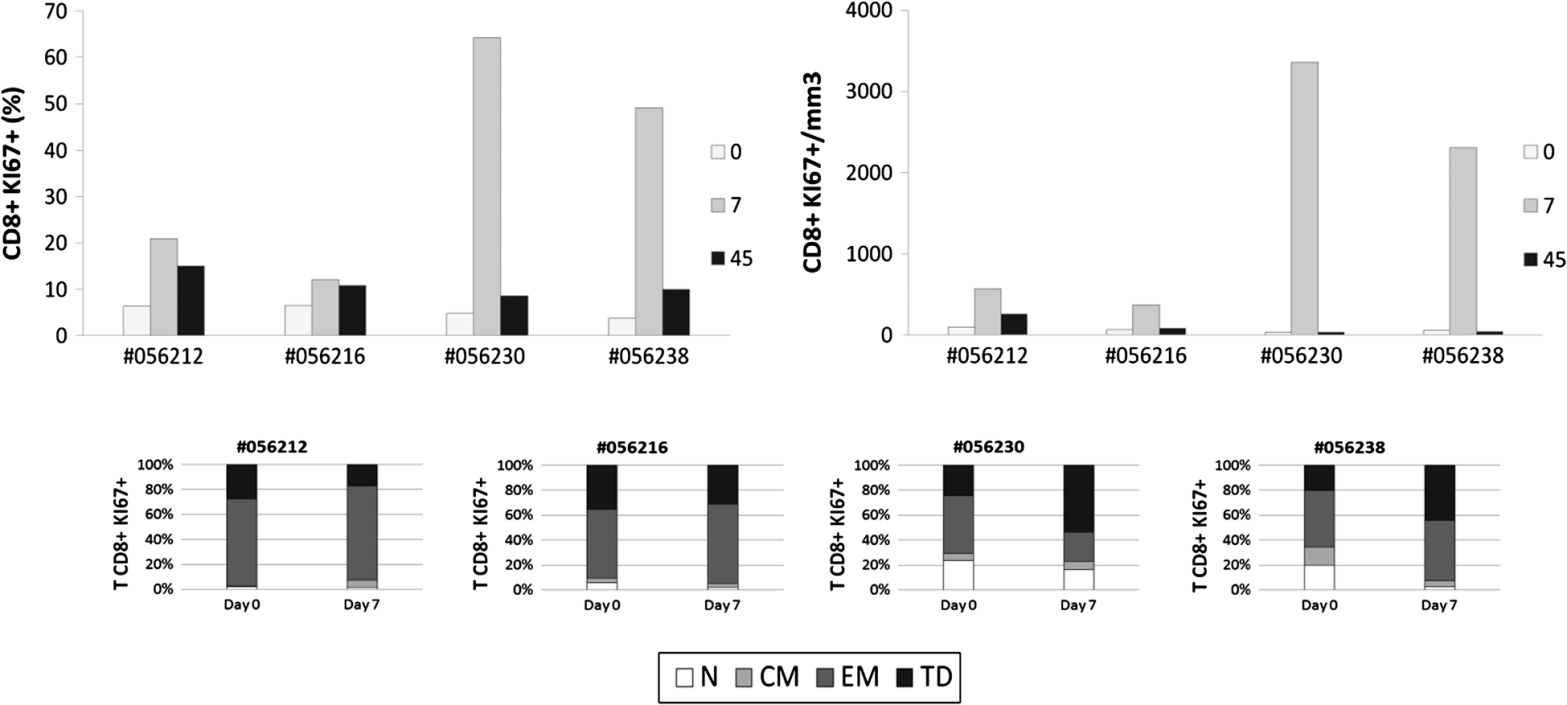
**Human rIL**-**2 transiently increases proliferationof CD8**^+ ^**T cell subset.** Ki67 expression was measured intracellularly in CD8^+^ T cells (top panel). Ki67 expression was then assessed in each CD8^+^ T cell subset (N, CM, EM or TD) at days 0 and 7 after IL-2 treatment (bottom panel). IL-2-treated macaques were #056212, #056216 (low dose) and #056230, #056238 (high dose).

Next, we evaluated the proportion of proliferating CD4^+^ and CD8^+^ T cells as assessed by the percentages of T cells expressing nuclear Ki67 Antigen (Figures 5 and 6).

This revealed a robust and transient rIL-2-associated increase in Ki67 expression in peripheral CD4^+^ (Figure 5 – top panel) and CD8^+^ T cells (Figure 6 – top panel). Consistent with the results obtained with global T cell counts, rIL-2 effect on proliferation seemed to be dose-dependent. Indeed, in animals treated with low rIL-2 dose, we observed an increase of Ki67-expressing CD4^+^ T cells from 17.7 and 6.8% before treatment to 25.7 and 12% at day 7 (1.4 and 1.7 fold increase respectively). Using high dose of rIL-2, Ki67 expressing CD4^+^ T cell population rose from 6.8 and 4.8% before treatment to 23 and 10.2% at day 7 (3.4 and 2.1 fold increase respectively) (Figure 5 – top panel).

Similarly, for CD8^+^ T cells, rIL-2 treatment transiently increased frequency of Ki67 expressing CD8^+^ T cells at day 7 by a 1,8 to 3,3 factor when used at low dose while this increase was up to 13 fold when animals were treated with high dose of rIL-2 (Figure 6 – top panel).

We then assessed cycling cells within each of the CD4^+^ and CD8^+^ T cell subsets before and at day 7 after treatment. As seen on Figure 5 (bottom panel), before treatment, the majority of cycling cells were effector memory CD4^+^ T cells. After treatment, Ki67-expressing EM CD4^+^ T cell subset was reduced by half and, consistent with the data obtained for the distribution of subpopulations, in low dose treated animals, the percentage of Ki67 expressing CD4^+^ T cells was increased in the central memory subset (Δ% = +54.8 and 32.5%) while in high dose treated macaques, this frequency was mostly augmented in the naïve subset (Δ% = +18.8 and 7%) and, to a lesser extent, in the central memory subpopulation.

Interestingly, in CD8^+^ T cells treated with low dose of rIL-2, the pool of cycling terminal differentiated cells decreased (Δ% = −10.6 and −4%) whereas it increased when using high dose (Δ% = +29.1 and 24%); rIL-2 having a modest impact on frequency of cycling EM cells compared to that observed in CD4^+^ T cells (Figure 6 – bottom panel).

These results showed that rIL-2 led to a transient increase of the proliferation and activation of CD4 and CD8 T cell subsets.

### Human rIL-2 modulates CD4^+^ and CD8^+^ T cell spontaneous apoptosis

**Figure 7:**
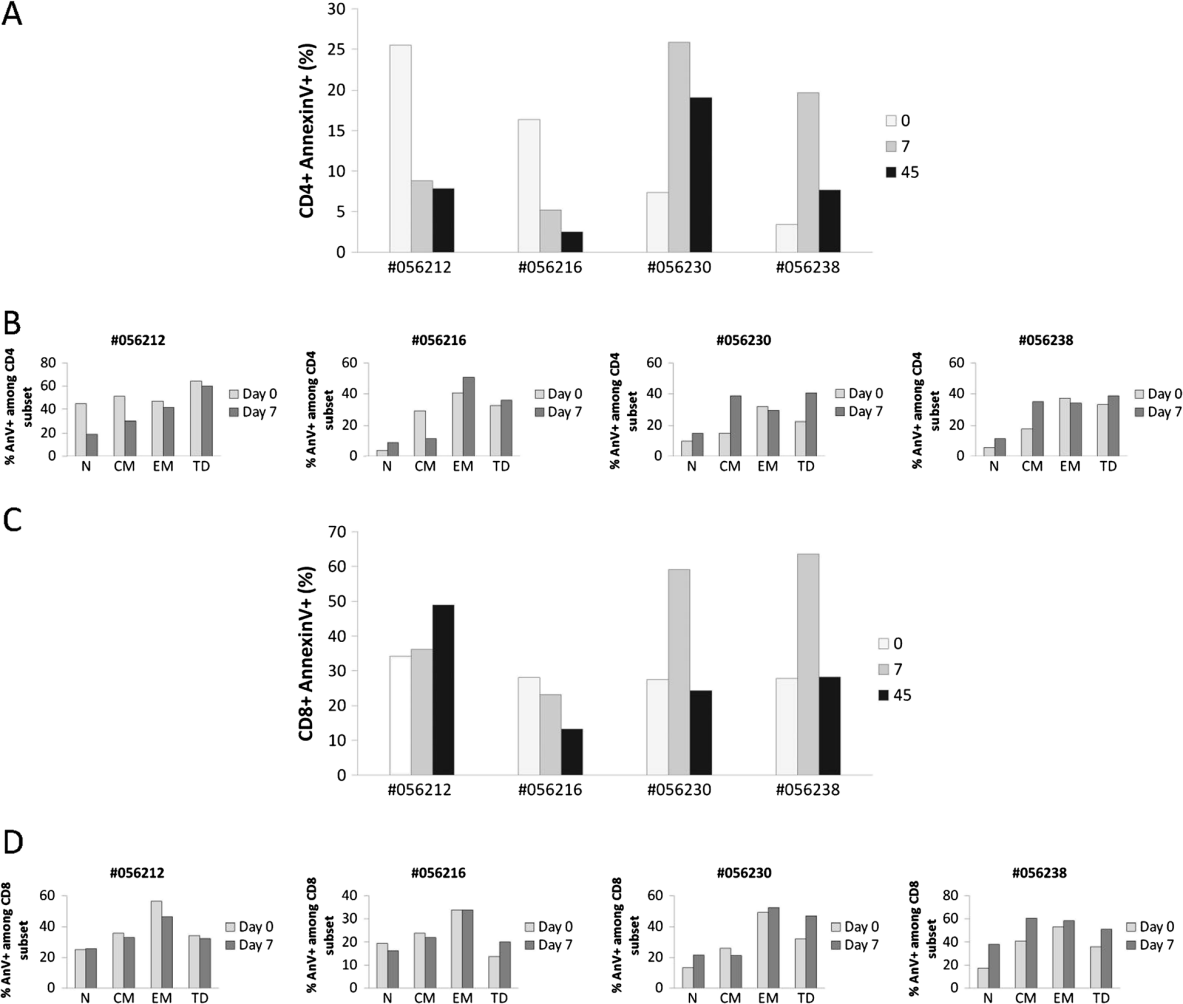
**Human rIL**-**2 modulates spontaneous CD4**^+ ^**and CD8**^+ ^**T cell apoptosis.** Apoptotic cell death was assessed using FITC-conjugated AnnexinV. Total CD4^+^ (**A**) and CD8^+^ (**C**) T cell apoptosis was measured after 16 h of culture at day 0, 7 and 45 after IL-2 treatment. In order to assess the propensity of each subset to undergo apoptosis, apoptosis was then measured in each CD4^+^ (**B**) or CD8^+^ (**D**) T cell subpopulation (N, CM, EM or TD) at day 0 and day 7 after IL-2 treatment. T cells, gated on lymphocytes gate followed by gating on CD4^+^ or CD8^+^ Tcells, were analysed for CD45RA and CD62L expression. Each subpopulation (N, CM, EM and TD) was then assessed for AnnexinV expression and sensitivity was calculated as follows for each subset:% *AnnexinV*^+^*among subset* = *N* (*CM*, *EM or TD*) *AnnexinV*^+^*T cells*/ (*N* (*CM*, *EM or TD*) *AnnexinV*^+^*T cells* + *N* (*CM*, *EM or TD*) *AnnexinV*^-^*T cells*) *x100*.

IL-2 has been shown to exert pro- or anti-apoptotic effects in a variety of hematopoietic cells. Furthermore, as we observed a transient activation of CD4^+^ and CD8^+^ T cells after treatment with rIL-2, followed by a contraction of effector memory and Terminally Differentiated T cell subsets, we wanted to analyze whether cell death could be induced in these populations. In this purpose, we evaluated CD4 and CD8 T cell apoptosis by quantifying cell surface expression of Phosphatidyl Serine residues by Annexin V staining (Figure 7).

Interestingly, our data revealed opposite effect of low versus high dose of rIL-2 on CD4^+^ T cells. Indeed, CD4^+^ T cells from monkeys treated with the lower dose were less prone to die than CD4^+^ T cells from macaques treated with the higher dose. As seen in Figure 7A, animals treated with low dose of rIL-2 showed a decrease in the frequency of apoptotic CD4^+^ T cells at day 7 compared with pre treatment values (Δ% = −16.7 and −11.2%) whereas high dose treated macaques exhibited a robust increase of CD4^+^ T cell apoptosis (Δ% = +18.5 and 16.3%). rIL-2 seemed to exert a long term effect on CD4^+^ T cell death as levels of apoptosis at day 45 remained either increased (Δ% = +11.7 and 4.3%) or decreased (Δ% = −17.7 and −13.9%) compared to basal levels in high or low dose treated animals respectively.

This dose-dependent effect on cell death was also observed in the CD8 T cell compartment as low dose rIL-2 had no effect on CD8 T cell apoptosis whereas high dose raised frequency of apoptotic CD8^+^ T cells by +31.5 and 35.8% in each animal, respectively. Unlike the effect on CD4 T cell compartment, this increase was transient (day 7) and frequencies of apoptotic cells returned to basal levels at day 45 (Figure 7C).

Given that rIL-2 impacted on T cell subsets distribution, we next analyzed susceptibility to undergo apoptosis within each CD4 or CD8 T cell subset. As seen in Figure 7B, rIL-2 mainly exerted its effect, either pro- or anti-apoptotic, on the central memory CD4^+^ T cell subpopulation. Indeed, in low dose treated animals, the frequency of apoptotic central memory CD4^+^ T cells decreased by a mean of 19.1% at day 7. In macaques treated with high rIL-2 dose, this frequency rose by a mean of 20.4%. Treatment with high dose of rIL-2 also induced an increase in naïve CD4 T cell death at day 7 after injection (from 9.7 and 5.6% to 15 and 14.4% - mean Δ% = 7%). In contrast to this specific effect on central memory CD4^+^ T cells, no difference on CD8 T cells apoptosis could be observed whatever the rIL-2 dose used (Figure 7D).

Thus, low dose of rIL-2 seemed to decrease sensitivity of central memory CD4^+^ T cells to spontaneous cell death while high dose of rIL-2 increased sensitivity of this population to undergo apoptosis. Moreover, while low dose of rIL-2 had no effect on CD8 T cell apoptosis, high dose clearly potentiated spontaneous CD8 T cell death. However, unlike its effect on CD4^+^ T cells, the cytokine did not specifically modulate sensitivity of any CD8 T cell subset but exerted a global effect on each CD8 T cell subpopulation.

### Human rIL-2 has a dose dependent effect on Fas-induced CD4 and CD8 T cell apoptosis

As we found that, depending on the dose of rIL-2 used, T cells were more or less sensitive to spontaneous apoptosis, we next sought to determine whether this differential sensitivity was also observed for Fas-induced cell death. Indeed, we and others have previously shown that Fas-mediated cell death is associated with progression to AIDS and chronic immune activation observed in HIV/SIV disease could drive T cells into apoptosis via Fas/FasL pathway [25-27].

First, we evaluated cell surface expression of CD95/Fas on CD4^+^ and CD8^+^ T cells. We showed that low dose of rIL-2 had almost no effect on CD95 cell surface expression either on CD4^+^ or CD8^+^ T cells. On the contrary, high dose of rIL-2 induced a low increase of CD95 expression at the surface of CD4^+^ and CD8^+^ T cells (data not shown).

**Figure 8:**
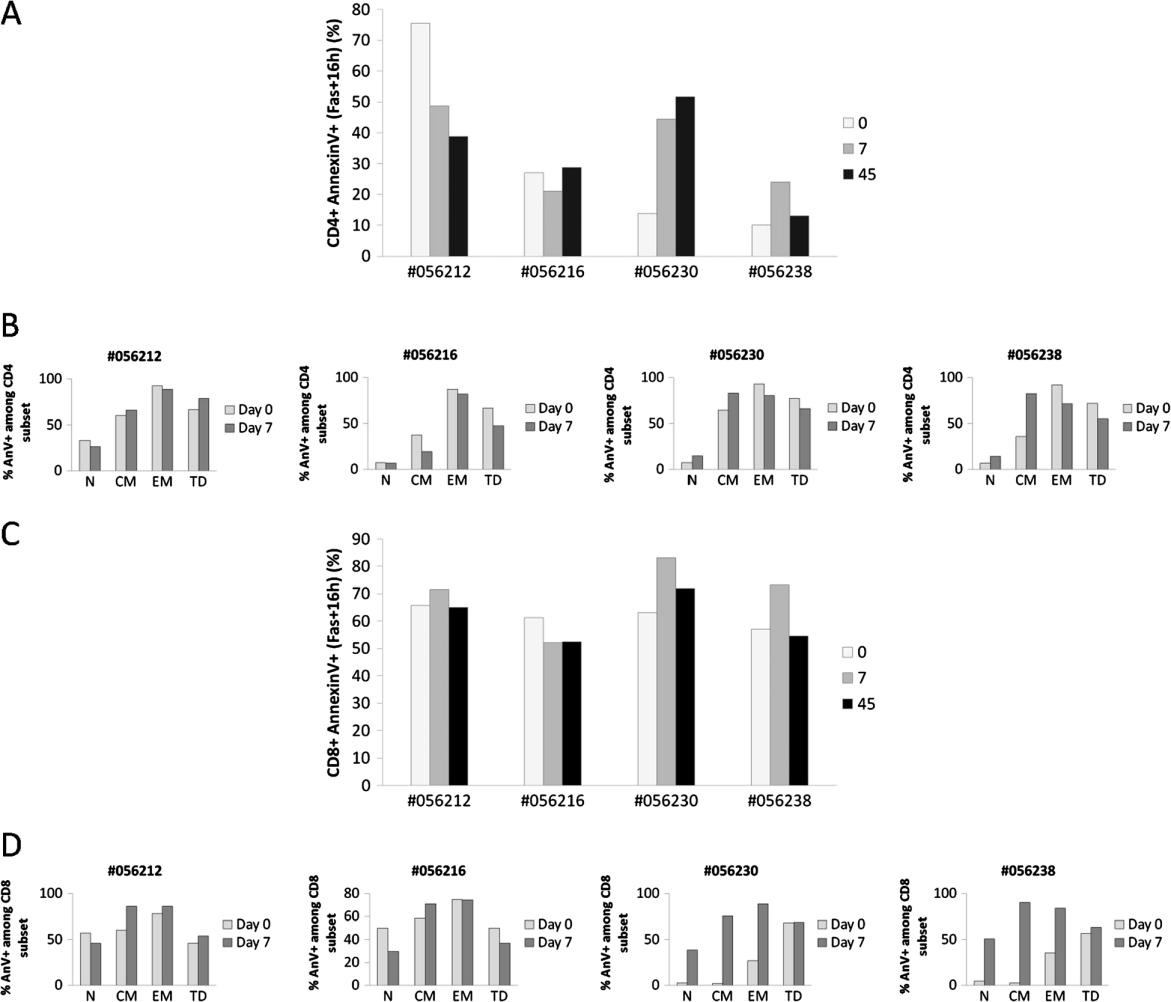
**Human rIL**-**2 modulates Fas**-**mediated CD4**+ **and CD8**+ **T cell apoptosis.** Fas-induced **a**poptotic cell death was assessed using FITC-conjugated AnnexinV. Total CD4^+^ (**A**) and CD8^+^ (**C**) T cell apoptosis was measured after 16 h of culture in the presence of FasL (200 ng/ml) at day 0, 7 and 45 after IL-2 treatment. In order to assess the propensity of each subset to undergo apoptosis, apoptosis was then measured in each CD4^+^ (**B**) or CD8^+^ (**D**) T cell subpopulation (N, CM, EM or TD) at day 0 and day 7 after IL-2 treatment. T cells, gated on lymphocytes gate followed by gating on CD4^+^ or CD8^+^ Tcells, were analysed for CD45RA and CD62L expression. Each subpopulation (N, CM, EM and TD) was then assessed for AnnexinV expression and sensitivity was calculated as follows for each subset:% *AnnexinV*^+^*among subset* = *N* (*CM*, *EM or TD*) *AnnexinV*^+^*T cells*/ (*N* (*CM*, *EM or TD*) *AnnexinV*^+^*T cells* + *N* (*CM*, *EM or TD*) *AnnexinV*^-^*T cells x100*.

As observed with spontaneous apoptosis, our results highlighted the dual effect of rIL-2 on Fas-induced CD4^+^ T cell apoptosis. Indeed, at day 7 after treatment, when animals were treated with low dose rIL-2, we observed a significant decrease in Fas-induced CD4^+^ T cell apoptosis (−26.7 and −6.1%) whereas when treated with high dose, animals experienced a robust increase in Fas-induced CD4+ cell death (+30.8 and +14.1%). At high dose, this rIL-2 proapoptotic effect seemed to be persistent as apoptotic rates were still increased at day 45 compared to baseline (Figure 8A).

Surprisingly, rIL-2 induced a more questionable effect on CD8+ T cell population as only high dose showed a reproducible effect in all animals with 20.1 and 16.2% increases in Fas-induced CD8+ T cell apoptosis at day 7 after treatment (Figure 8C).

Thereafter, we studied sensitivity of each subset to Fas-induced apoptosis. As shown on Figure 8B, treatment with low dose of rIL-2 induced an overall slight effect on every CD4 T cell subsets (from −3.5 to −6% depending on the subset) whereas treatment with high dose of rIL-2 led to a more contrasting effect. Indeed, it increased sensitivity of naïve (Δ% = +7.5 and +7.4%) and central memory (Δ% = +18.5 and +46.8%) subsets while it decreased sensitivity of effector memory (Δ% = −12.7 and −20.6%) and terminal differentiated (Δ% = −11.3 and −16.7%) subsets (Figure 8B). Regarding CD8^+^ T cells, we showed that treatment with low dose of rIL-2 decreased sensitivity of naïve CD8^+^ T cells to Fas-induced apoptosis (Δ% = −11.3 and −20.2%) whereas it increased sensitivity of CD8 central memory subset (Δ% = +26.1 and +12.7%) (Figure 8D). When animals were treated with high dose of rIL-2, a dramatic increase in sensitivity to Fas-induced apoptosis was observed in CD8 naïve (Δ% = +36.2 and +45.6%), central memory (Δ% = +73.3 and +87.4%) and effector memory (Δ% = +61.5 and +48.8 %) subsets (Figure 8D).

Thus, we showed here that treatment with rIL-2 could modulate Fas-induced cell death. This modulation was particularly obvious when rIL-2 was used at high dose with a strong potentiator effect on Fas-induced CD4 central memory and CD8 naïve, central and effector memory T cells apoptosis.

## Discussion

Both enhanced T cell turnover and survival have been implicated in regulating the size of the peripheral T lymphocyte pool following IL-2 immunotherapy in HIV-infected patients. To date, selective expansion of CD4^+^ T cells is considered as the hallmark of IL-2-driven immune reconstitution. According to the latest data, the increase of CD4+ T cell count due to IL-2 immunotherapy in ART treated patients does not confer clinical benefit but may induce grade 4 clinical side effects [24]. Several hypotheses were evoked to explain these disappointing results. IL-2 side effects might overcome beneficial effects on host defenses or the CD4+ T cells induced by IL-2 treatment may fail to exert protective effector functions. Based on these observations, we wanted to explore the impact of IL-2 immunotherapy in a model of infection (i.e.: SIV infection of Rhesus macaques) without ART treatment in order to observe only IL-2-induced changes on various immunological parameters.

First studies using IL-2 therapy in HIV patients had shown that this treatment could induce transient increases in HIV viremia [28] at least due to higher levels of CCR5 expression [16]. However, other studies had reported that most of IL-2 treated patients under ART showed no increase in plasma viral load [29,30]. Here, although rIL-2 treatment leads to a dramatic increase of CD4 T cell subset (including cycling cells (Ki67^+^)), plasmatic viral load is not changed in ART naïve SIV-infected monkeys. These results are consistent with data previously obtained in our laboratory [30] and with the UK Vanguard study which showed that intermittent cycles of subcutaneous IL-2 produced significant increases in CD4 T cell counts without affecting viral load in ART-naïve HIV-infected patients [31]. Thus, according to our results, adverse IL-2 effects do not seem to be due to its effect on HIV viremia, even in the absence of ART treatment. Therefore, we addressed several questions to help explaining the lack of clinical benefit observed in SILCAAT/ESPRIT studies. First, we studied the effect of IL-2 on CD4 and CD8 subpopulations. Then, we wanted to confirm and extend results previously obtained in our group [23] concerning IL-2 driven Treg induction. Finally, given the controversial literature concerning IL-2 and apoptosis, we wanted to clarify the role of this cytokine on CD4 and CD8 T lymphocyte cell death *in vivo*.

First, our data show that treatment of chronically SIV-infected Rhesus macaques with rIL-2 induced a rapid, transient, dose-dependent increase in peripheral T cell counts at day 7 after treatment. This immunotherapy not only induces CD4^+^ T cell expansion but also affects CD8^+^ T cell pool early after treatment. These results are consistent with data obtained following IL-2 immunotherapy in metastatic melanoma patients in which levels of perforin and granzyme - which are essential for CD8^+^ T cell cytotoxicity - were increased [32]. Since then, many other studies have shown that IL-2 immunotherapy mediated tumor regression via enhanced endogenous tumor specific CD8^+^ responses [33,34]. Nevertheless, a dramatic CD4 lymphopenia occurred at day 45 in all treated animals while CD8^+^ T cell loss only occurred in high dose treated animals.

We demonstrate that treatment with low or high dose of rIL-2 has a differential effect on CD4 and CD8 T cell subpopulations repartition. Indeed, regarding CD4 T cell subsets, the higher dose of rIL-2 selectively expanded naïve and central memory compartments whereas low dose only affected memory populations (central and effector). These rIL-2 selectively expanded CD4^+^ T cells are activated and express proliferation markers. These cells are most likely new thymic emigrants as it was strongly suggested that a naive T-cell increase reflected thymic export after IL-2 therapy in HIV-infected patients [35]. In addition, while low dose seemed to exert a transient effect, higher dose induced a sustained effect as changes observed in subsets distribution were maintained at day 45 after treatment. Finally, both doses induced a drastic depletion of terminally differentiated CD4^+^ T cells (at day 45 after treatment) associated with the occurrence of a wasting syndrome characterized by weight loss and diarrhea. These results are consistent with data obtained in the UK-Vanguard trial which suggested that after IL-2 discontinuation, CD4 T cell counts declined rapidly even below therapeutic threshold [36]. We therefore propose that although rIL-2 contributes to correct the HIV-driven unbalance within naïve and central memory CD4^+^ T compartments, it also leads, in the absence of antiretroviral treatment, to T cell exhaustion and immune failure through excessive expansion, proliferation, activation and apoptosis of CD4 naïve and central memory subsets.

IL-2 is well-known as a key regulator of immune tolerance as it promotes the development and peripheral expansion of CD4^+^ CD25^high^ Tregs. In the context of HIV, several reports have suggested that Tregs may have a beneficial role either by suppressing viral replication or by limiting non specific activation [37,38]. On the contrary, other studies suggested that Tregs may account for premature and persistent suppression of effector response, thus contributing to establishment of a chronic disease state. Indeed, Tregs have been shown to inhibit HIV-specific T-cell responses *ex vivo* and may act *in vivo* by suppressing HIV-specific immunity, allowing HIV to persist [39-42]. We have recently reported that, following IL-2 immunotherapy, resting and activated Tregs were expanded in HIV-infected patients under HAART regimen [23]. They suggest that these CD4^+^ Tregs may inhibit the generation of responses against pathogens thereby providing a beginning of explanation for the disappointing results of ESPRIT and SILCAAT studies. We largely confirm and extend these results in our model as rIL-2, at low dose, drives a massive, transient peripheral CD4 Treg expansion. At day 45, however, size of the CD4 Treg compartment returns to baseline value (low dose treatment) or is even decreased compared to baseline (high dose treatment). This decrease in peripheral blood may reflect relocation of CD4 Tregs in lymphoid tissues, particularly in the gut [43], thus strengthening loss of the Th17/Treg cell balance that is already observed in progressive HIV/SIV infection associated with the expression of TGF-β [44-47]. Concerning Treg populations, we also show that treatment with rIL-2 induces a sustained and dose-dependent peripheral expansion of a poorly studied population, namely CD8^+^ Tregs. These results are consistent with the study of Nigam *et al*. who recently demonstrated that peripheral CD8^+^CD25^high^FoxP3^+^ T cells were induced early after SIV infection and that the number of these cells was positively correlated with viral load and associated with a poor prognosis. This rapid expansion of CD8 Tregs in the blood was also observed in multiple lymphoid tissues of SIV-infected Rhesus macaques but not in non pathogenic SIV infection of Sooty Mangabeys. Moreover, they showed that these cells were able to suppress antiviral immunity thereby suggesting a deleterious role for this CD8^+^ T cell subset during the course of the disease [48]. In addition, following treatment with rhIL-2, a dramatic increase in CD8^+^ Foxp3^+^ T cell prevalence was observed in the circulation and tumor-draining lymph nodes of subcutaneous tumor-bearing mouse models [49]. Effect of rIL-2 on the T regulatory compartment thus provides a first piece of explanation for the rapid development of wasting syndrome in our animals. Indeed, rIL-2 may act, on the one hand, on CD4 Treg population, inducing its expansion and relocation to gut-associated lymphoid tissues (GALT) thereby contributing to loss of Th17/Treg balance. On the other hand, rIL-2 provokes peripheral expansion of CD8^+^ Treg subset contributing to defective antiviral immunity.

Finally, our study underlines the dual role of IL-2 in the context of spontaneous and Fas-induced apoptosis. Several groups have already investigated the effect of IL-2 immunotherapy on apoptosis but results were quite discordant. Indeed, it was shown that, in HIV-infected patients, IL-2 treatment was associated with increased apoptosis rates [50]. On the opposite, some studies showed that IL-2 could reduce lymphocyte apoptosis in HIV infected patients [51]. Here, we report that IL-2 can effectively play both role depending on the dose used, high dose having a proapoptotic effect on both CD4^+^ and CD8^+^ T cells and low dose being antiapoptotic in CD4^+^ T cells only. This modulating effect on cell death seems to be restricted to CD4^+^ central memory T cells while it exerts a global effect on each CD8^+^ T cell subsets. Excessive apoptosis observed in high dose treated animals could be due to higher expression of Fas receptor on CM CD4^+^ T lymphocytes cell surface, indeed we observed that Fas expression on CD4^+^ T lymphocytes was increased after rIL-2 treatment (data not shown). We and others have reported that T cells from HIV-infected individuals or SIV-infected macaques are highly prone to undergo apoptosis after Fas-ligation [6-8,27]. A recent study showed that Rhesus macaques treated with neutralizing anti-FasL antibody preserved higher levels of central memory CD4^+^ T cells [52] thereby supporting a critical role for Fas in the CD4^+^ T cell compartment. Herein, we found that *in vivo* IL-2 treatment has a detrimental effect on the susceptibility of cells to undergo apoptosis after Fas ligation. However, we cannot exclude a role for other death molecules like PD-1 or TRAIL that have been proposed to participate in the death of T cells during HIV/SIV infection [46,53-56]. Further investigation of these cell death pathways among various lymphoid populations should provide new insights about immunological mechanisms implicated in IL-2 immunotherapy.

## Conclusions

Following rIL-2 treatment in SIV-infected Rhesus macaques, T cells - including CD4 and CD8 Tregs - are therefore not only expanded but, in addition, become more susceptible to undergo apoptosis providing a rational for disease progression. Thus, our data contribute to the understanding of the homeostatic and dosage effects of IL-2 in the context of SIV/HIV infection. Although the conclusions drawn in SIV monkey models are not easy to translate to human infected with HIV, we believe that our data provide new insights in this field regarding the important and long term commitment in developing IL-2 therapy.

## Methods

### Animals

Rhesus Macaques (*Macaca mulatta*) of Chinese origin were inoculated intravenously with 10 AID_50_ (50% animal infectious dose) of the SIVmac251 strain. All the animals were challenged with the same batch of virus (provided by AM. Aubertin, INSERM U74, Strasbourg, France), titrated *in vivo* in RMs, and stored in liquid nitrogen. Animals were seronegative for simian T leukemia virus type 1, SRV-1 (type D retrovirus), herpesvirus B, and SIVmac. Animals were housed and cared for in compliance with existing French regulations (Institut Pasteur, Paris, France). All the animal experiments described in the present study were conducted at the Institut Pasteur according to the European Union guidelines for the handling of laboratory animals ( http://ec.europa.eu/environment/chemicals/lab_animals/home_en.htm). The protocol was approved by the committee on the ethics of animal experiments of Ile de France. All surgery was performed under sodium pentobarbital anesthesia, and all efforts were made to minimize suffering. Macaques in chronic phase of infection were then either treated with 6.10^5^ UI/kg body weight (high dose), 1.4.10^5^ UI/kg body weight (low dose) recombinant human IL-2 (Macrolin® Aldesleukine rhIL-2) or saline solution (control) daily for 5 days. Blood samples were collected before IL-2 treatment (day 0) and at day 7, 10, 15, 30 and 45 after treatment.

### Lymphocyte immunophenotyping and flow cytometry

Blood from monkeys was collected in sterile EDTA-treated vacuum tubes and cell populations were analyzed by flow cytometry using the following antibodies: anti-CD20-FITC (clone 2 H7), anti-CD4-PE (clone L200), anti-CD62L-FITC (clone SK11), anti-CD62L-PE (clone SK11), anti-CD25-PE (clone M-A251), anti-CD3-PerCP (clone SP34-2), anti-CD4-PerCP (clone SK3), anti-human CD8-PerCP (clone SK1), anti-CD8-APC (clone RPA-T8), anti-CD95-APC (clone DX2) and anti-CD127-Alexa 647 (clone HIL-7R-M21) were purchased from BD Biosciences/Pharmingen (San Jose, CA). Anti-CD45RA-APC (clone T6D11) was obtained from Miltenyi (Bergisch Gladbach, Germany). For intracellular staining, the cells were fixed and permeabilized before incubation with anti-Ki67-FITC (clone Ki67, Dako, Glostrup, Denmark), or anti-Fox-P3-Alexa 488 (clone 259D/C7, BD Pharmingen, San Jose, CA).

Cells were analyzed by flow cytometry. A total of 30000 events in the lymphocyte gate were analyzed using CellQuest software (BD Biosciences). All analyses were performed with a FACScalibur flow cytometer (BD Biosciences).

### Cell death quantification

PBMCs from monkeys were isolated from peripheral blood by density gradient centrifugation LymphoPrep (PAA Laboratories, Pasching, Austria) and cultured in RPMI 1640 supplemented with 10% FCS, 1% glutamine, 1% pyruvate, and 1% antibiotics. Up to 500,000 PBMCs were cultured overnight in the absence or presence of recombinant Fas-L (10 μg/ml, Alexis Corporation). Cell death was assessed by flow cytometry, as previously described [8]. Briefly, after staining with specific antibodies (30 min at 4°C), cells were washed and then incubated with FITC labeled Annexin-V (10 min at 4°C), and 30000 events were analyzed with a FACScalibur flow cytometer (BD Biosciences).

### Viral quantification

RNA was extracted from plasma of SIV-infected monkeys using the TRI Reagent BD Kit (Molecular Research Center, Cincinnati, OH). Real-time quantitative RT-PCR was used to determine viral loads as previously described [57].

## Competing interests

The authors declare that they have no competing interests.

## Authors’ contributions

JG carried out the experimental work and wrote the paper. ML carried out the viral quantification experiments. RS, SM and LCG helped with the monkeys. YL and JE conceived the study, oversaw its execution and edited the manuscript. All authors read and approved the final manuscript.

## Funding information

This study was supported by research funding from the ANRS to JE and YL and the “Fondation pour la Recherche Médicale”, to JE. JG and ML were supported by fellowships from ANRS, SM by SIDACTION and RS by a fellowship from the FCT program Ciência 2008.

## Supplementary Material

Additional file 1 **Human rIL-2 does not affect hematological parameters.** Four Rhesus macaques were infected with the pathogenic SIVmac251 strain and then treated during chronic phase with human rIL-2 (low dose: ○,Δ or high dose: ●,▴). Each symbol represents one individual. Hematological and physiological parameters such as platelet erythrocyte counts, hematocrit, temperature, weight were measured at the day of treatment and thereafter. Click here for file
