# Supplementary figures and images for: IL-2 immunotherapy in chronically SIV-infected Rhesus Macaques
Source: Virol J. 2012 Sep 28;9:220. doi: 10.1186/1743-422X-9-220 (PMC3499432; doi:10.1186/1743-422X-9-220)

Supplemental Data

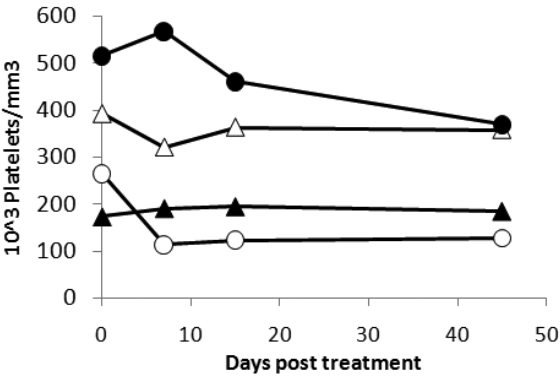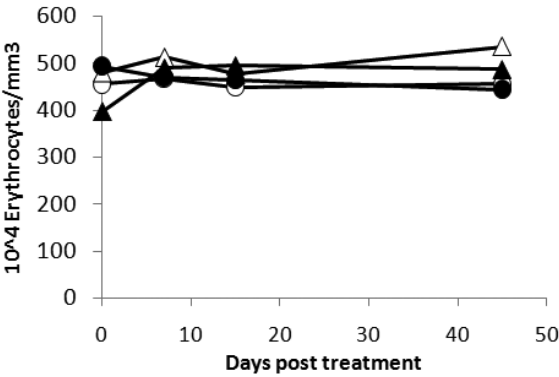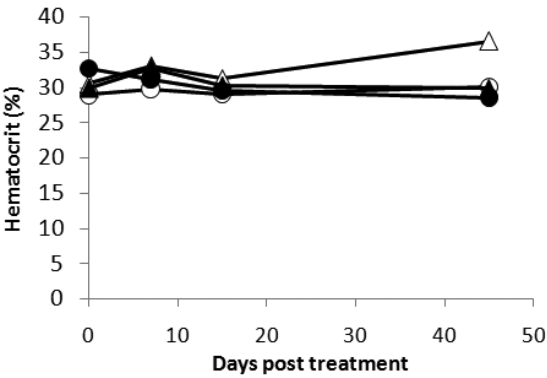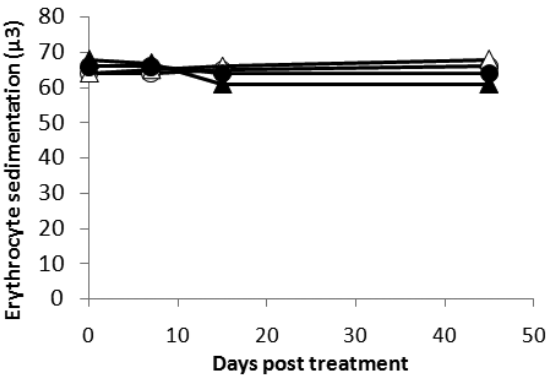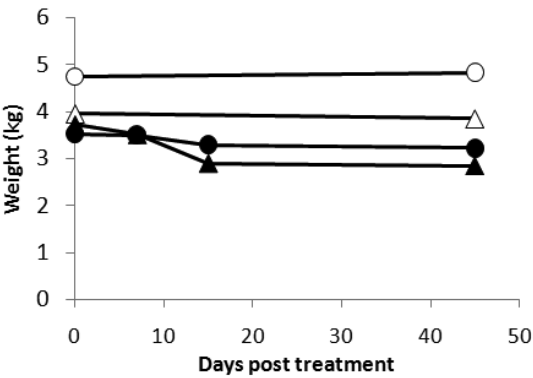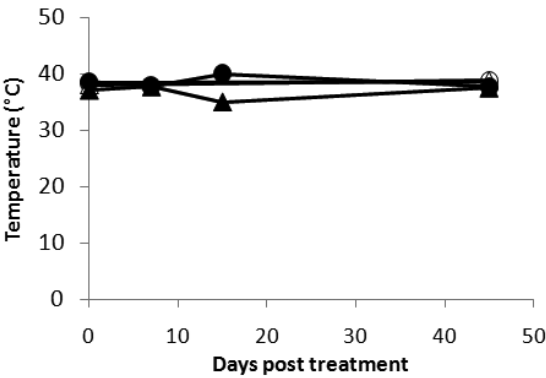

Supplement: Additional file 1 — Human rIL-2 does not affect hematological parameters. Four Rhesus macaques were infected with the pathogenic SIVmac251 strain and then treated during chronic phase with human rIL-2 (low dose: ○,Δ or high dose: ●,▴). Each symbol represents one individual. Hematological and physiological parameters such as platelet erythrocyte counts, hematocrit, temperature, weight were measured at the day of treatment and thereafter. [file 1743-422X-9-220-S1.pdf]
